# Self-organization of virtual odorant receptors inspired by insect olfaction

**DOI:** 10.1186/1471-2202-12-S1-P286

**Published:** 2011-07-18

**Authors:** Amir Madany Mamlouk, Michael Schmuker

**Affiliations:** 1 Institute for Neuro- and Bioinformatics, University of Lübeck, Lübeck, Germany; 2 Neuroinformatics & Theoretical Neuroscience Department, Freie Universität Berlin, Germany; 3 Bernstein Center for Computational Neuroscience Berlin, Berlin, Germany

## 

Schmuker & Schneider [1] proposed a system of virtual receptors to mimic insect olfaction with a simple network model. In this model, a set of 836 odorants described by 184 chemical properties is used to span a virtual chemical space. A set of virtual receptors, i.e. codebook vectors moving in this space, is then trained using a self-organizing map [2], mimicking sensor evolution with the assumption that the receptors are tuned to represent the chemical space as good as possible. It has been shown that this architecture is less sensitive to the network size when using lateral inhibition as a pattern processing method. Furthermore, in this work the activity patterns show a nice similarity to well-known experimental recordings of bulbar activity in rodents [3].

Historically, the idea of SOMs is biologically motivated by the topology conserving structure as it is well-known for the somatotopic organization of the cerebrum. On the other hand, if the data really is high-dimensional, i.e. the intrinsic dimension is >>2D – unlike the almost 2D organization of the skin – then we will have strong topological defects on the map. In this case, the chemical space is 184-dimensional (the number of properties used) – far more complex than the 2D SOM. SOMs have the benefit that they can learn topologies in high-dimensions and still are very easily displayed in 2D, as all the virtual receptors are lying independently of the input space in a fixed 2D-grid. But without doubt, better visibility is not a fitness criterion likely to be found in the chemotopic evolution of odorant receptors.

Other topology conserving algorithms are known to be much better suited to learn such high-dimensional topologies, like e.g. Neural Gas [4], a soft-competitive learner that learns the chemical space position of the virtual receptors similar to the SOM. But the neighborhood relations are not based on a fixed receptor grid but rather dependent on the receptor similarities in the chemical space. The net’s connectivity during sensor evolution is induced using a Hebbian-like learning of co-activation by the virtual receptors.

We will discuss in this work the differences of the NG-tuned compared against the SOM-tuned virtual receptors for the following aspects: classification performance, topology conserving properties, scalability of the architecture, and the effect of insect-like pattern processing by lateral inhibition. Just the same, we propose that NG is better suited to learn the topology, i.e. mimic the evolutionary tuning of the virtual receptors, while SOMs are very useful in providing a visual interface for bulbus-like display of odorant stimulation images.

As an outlook, we want to motivate how one could check not only for topological defects in the given chemical space but also check for experimental data. E.g. is there a correlation between experimental uptake images as published by Johnson & Leon [3] and one of the proposed sets of virtual receptors? Is there something we can learn about the tuning strategies of the olfactory system?
